# The lipid composition of extracellular vesicles: applications in diagnostics and therapeutic delivery

**DOI:** 10.3389/fmolb.2023.1198044

**Published:** 2023-07-13

**Authors:** Samaneh Ghadami, Kristen Dellinger

**Affiliations:** Department of Nanoengineering, Joint School of Nanoscience and Nanoengineering, North Carolina A&T State University, Greensboro, NC, United States

**Keywords:** extracellular vesicles, lipidome, exosomes, targeting, exosome engineering, lipids, Alzheimer’s disease, cancer

## Abstract

Extracellular vesicles (EVs), including exosomes, with nanoscale sizes, biological origins, various functions, and unique lipid and protein compositions have been introduced as versatile tools for diagnostic and therapeutic medical applications. Numerous studies have reported the importance of the lipid composition of EVs and its influence on their mechanism of action. For example, changes in the lipidomic profile of EVs have been shown to influence the progression of various diseases, including ovarian malignancies and prostate cancer. In this review, we endeavored to examine differences in the lipid content of EV membranes derived from different cell types to characterize their capabilities as diagnostic tools and treatments for diseases like cancer and Alzheimer’s disease. We additionally discuss designing functionalized vesicles, whether synthetically by hybrid methods or by changing the lipid composition of natural EVs. Lastly, we provide an overview of current and potential biomedical applications and perspectives on the future of this growing field.

## 1 Introduction

Extracellular vesicles (EVs) are a category of lipid-bound particles that can be produced and released by various cells (Simpson et al., 2008). They are typically classified into three subgroups: exosomes, ectosomes, and apoptotic bodies. The smallest subgroup (∼30–150 nm) are exosomes, characterized by lipid bilayer membranes and typically spherical or cup-shaped morphologies. Initially, it was believed that EVs functioned exclusively in cellular debris degradation, but the molecular characterization of the EV membrane, which can consist of various proteins (e.g., Rab GTPases, annexins, flotillin, ALG-2-interacting protein X, and tumor susceptibility gene 101 protein) and lipids (e.g., cholesterol, sphingolipids, ceramide, and glycerophospholipids) lent itself to the idea that EVs might be far more versatile in their mechanism of action. Indeed, these multifunctional structures were shown to carry different molecules (e.g., DNA, RNA, proteins, lipolytic enzymes, miRNA) for various purposes to specific destinations (Simons and Raposo, 2009; Record et al., 2014; Conlan et al., 2017; Jiang et al., 2019). Their unique structure also means that EVs can transport lipids such as sphingomyelin, cholesterol, lysophosphatidylcholine, arachidonic acid, other fatty acids, prostaglandins, and leukotrienes either within their membrane or lumen to diverse locales (Record et al., 2014).

Many reasons underpin the importance of characterizing the composition and role of lipids in EV interactions. This includes the involvement of EV lipids in disease pathogenesis, such as cancer (Sagini et al., 2018; Wang et al., 2022), as well as inflammation and immunity (Showalter et al., 2019). For example, exosomes released from T cells were found to be enriched in cholesterol and phosphatidylserine and can induce cholesterol accumulation in monocytes, which leads to the production of tumor necrosis factor-α (TNF-α) (Zakharova et al., 2007). A potent proinflammatory cytokine, this increase in TNF-α can result in necrosis or apoptosis. Indeed, Skotland *et al.* published a review article highlighting the state-of-the-art in exosomal lipid research covering different cell lines and biological fluids, such as seminal fluid, urine, and even nematodes. The paper provides an insightful overview of supporting evidence regarding the influence of lipids on exosome behavior (Skotland et al., 2019) and outlines important knowledge gaps related to the lipidomic component of exosomes, such as lipid function and the biology of EVs, supporting the need for more research in this field.

Lipids have diverse functional roles in the body, influencing different stages of exosomal biogenesis and altering metabolism and distribution. With lipids being a key component of EVs composition, they have been shown to increase cholesterol accumulation in the brain, liver, and spleen and, thereby have been associated with cholesterol-related storage diseases (Record et al., 2014). At the same time, their role in regulating lipid metabolism in adipocytes is due to lipidic enzymes, such as phospholipases (Record et al., 2014). Based on an analysis by Donoso-Quezada *et al.*, the number of publications related to EVs/exosomal lipids indexed in PubMed is steadily growing; however, the number is still significantly lower compared to proteomic analyses. In this review, we discuss the role of lipids in EVs, focusing on how lipid changes in EV membranes may impact pathological conditions and highlight their potential use as biomarkers. We additionally introduce concepts and approaches used to functionalize exosomes by changing the lipid composition and discuss applications in diagnostic and therapeutic fields, focusing on neurodegenerative disease.

### 1.1 Extracellular vesicle lipid composition

EVs of different cellular origins have membranes containing various distributions of lipids. For example, studying lipids in PC-3 cells showed that the lipid compositions of exosomes differ from their parent cells (Kanninen et al., 2016) (Skotland et al., 2019) (Record et al., 2014).

In general, based on lipid composition studies performed on EVs of different cell types (PC-3, Oli-neu, clonal derivative of hepatocellular carcinoma, B-lymphocytes, mast cells, dendritic cells, reticulocytes, platelets, and adipocytes), cholesterol constitutes the highest amount then phosphatidylcholine. The exception is for B-lymphocytes and PC-3 cells, which have higher sphingomyelin than phosphatidylcholine in their exosomes (Skotland et al., 2019). Exosomes released from Oli-neu cells (Trajkovic et al., 2008) have a higher level of ceramides and a lower level of sphingomyelins compared to exosomes from PC-3 cells (Llorente et al., 2013). Mass spectrometry analysis of exosomes released from PC-3 cells shows similar enrichment of lipids like cholesterol, long-chain sphingolipids, and phosphatidylserine 18:0/18. Still, higher enrichment of glycosphingolipids (hexosylceramide and lactosylceramide) in the exosome membrane compared to their parental PC-3 cells (Llorente et al., 2013).

Several additional studies have examined EV lipid composition using asymmetric flow field-flow fractionation. For example, exosomes from breast cancer cell lines (MDA-MB-231) and murine melanoma cell lines (B16-F10 cells) showed 80%–90% phosphatidylcholine (cholesterol was not included in measurements), and exosomes from pancreatic cancer cells (AsPC-1 cells) were shown to have 60% phosphatidylcholine and 30% sphingomyelin (Zhang et al., 2018). Comparing lipid enrichment from cells to exosomes in U87 glioblastoma cells, Huh7 hepatocellular carcinoma cells, and human bone marrow-derived mesenchymal cells in one study showed a high level of cholesterol and no sphingomyelin in Huh7 and mesenchymal cells; while the opposite ratio was seen in U87 cells (Haraszti et al., 2016). There was a high similarity between the lipid profile of Huh7 and mesenchymal stem cells. In the same study, exosome enrichment of lyso-derivatives (hydrolyzed lipids) of phosphatidylserines, phosphatidylglycerols, and phosphatidylinositols was observed in mesenchymal stem cells and Huh7 cells. Generally, a relationship was found to exist between lipid enrichment in extracellular vesicles and the head group charge, fatty acid tail length, and saturation (Haraszti et al., 2016).

Lipid arrays have even been shown to change between EVs with different sizes. For example, using asymmetric-flow field-flow fractionation, Zhang et al. (2018) classified the exosomes of B16-F10, MDA-MB-4175, and AsPC-1 into different subtypes based on their size, including large exosomes (Exo-L; 90–120 nm), small exosomes (Exo-S; 60–80 nm) and a smaller non-membranous group called exomeres (∼35 nm). They found that phosphatidylcholine composed 46%–89% of the lipid components in all exosomes. Lower levels of sphingomyelin (2%–10%) were recorded, except for AsPC-1 exomers which had a higher level of diglyceride (38%) and triglyceride (26%); while AsPC-1 Exo-S/L had higher levels of sphingomyelin (28%). This study is particularly notable because of this interesting relationship between exosomal lipid composition and size. The results indicate that lipid diversity changes exist in exosomes based on size and that it may be relevant to consider multiple exosome properties to increase the accuracy/applicability of published data.

Diverse lipidomic arrays have also been observed in EVs derived from the nervous system, including neural stem cells, neurons, astrocytes, microglia, oligodendrocytes, Schwann cells, and endothelial cells (Fauré et al., 2006; Sharma et al., 2017). For example, exosomes released from both neuronal and glial cells were found to be enriched with ceramides and glycosphingolipids (gangliosides) (Czubowicz et al., 2019). In addition, exosomes derived from mouse neuroblastoma (N2a) cells showed a high level of cholesterol, sphingomyelin, and ceramides compared to their parental cells (Yuyama et al., 2014). Interestingly, a comparison of four lipid categories (glycerophospholipids, sphingolipids, glycerolipids, and sterol lipids) between the frontal cortex tissue and brain-derived EVs using semi-quantitative mass spectrometric analysis showed that brain-derived EVs had higher glycerophospholipids, and lower sphingolipids (Su et al., 2021). High enrichment of glycerophosphoserine and low levels of glycerolipids and cholesterol ester lipids were also observed. Based on this work, we may conclude that different areas of the brain produce exosomes with unique lipid compositions, which may affect function and fluidity, and furthermore, may be of interest to consider as a potential area for neural targeting of agents or study of neurodegenerative diseases.

Overall, from these analyses, it is clear that lipid sorting and enrichment in the EV membrane differs for each cell line and cell type and that these are important factors to consider in exosome production, especially when studying the role of a specific lipid. For example, N2a cells are an excellent choice for selecting exosomes with high levels of ceramides. As more work is done in the field of exosomal lipids, standardized protocols are needed (such as using exosomes isolated only by a specific technique or lipid analysis method) to comparable published studies and generate reliable data. In addition, what is yet to be uncovered is unraveling how the complexity of these differences impacts function. This will help answer questions about how these variations in lipid compositions in different cell lines can be harnessed to influence behavior, be used as biomarkers for disease or other applications in the biomedical space.

### 1.2 The role of extracellular vesicle membrane lipids

**FIGURE 1:**
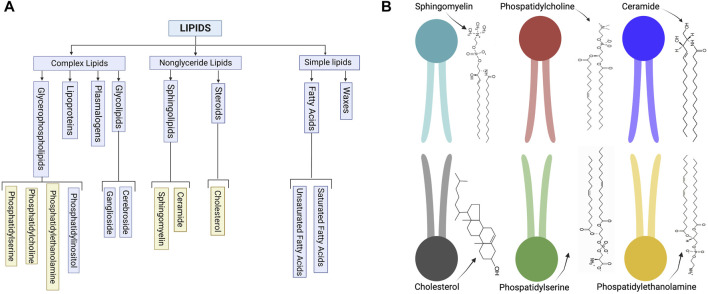
**(A)** Schematic of common lipid classifications. **(B)** Schematic of common lipid structures in the bilayer membrane of EVs (created with BioRender.com).

Several lipid types are found in EV membranes, though we can classify them into select categories (Figure 1A) based on their structure (Figure 1B). These classifications include steroids, sphingolipids, and glycerophospholipids (Kalra et al., 2012; Skotland et al., 2019). Adding a layer of complexity, each cell type produces exosomes with unique and distinct lipid compositions, which are asymmetrically arrayed in the EV membrane (Skotland et al., 2019). For example, some lipids, such as phosphatidylserine, usually assemble in the inner leaflet of the membrane, while others, such as very long chain sphingolipids and phosphatidylcholine, are arranged in the outer leaflet. Enzymes, such as flippases, floppasses, and scramblases, can mediate the translocation of lipids between two leaflets. The lipid structure accordingly affects the EV membrane features (Skotland et al., 2019).

**FIGURE 2:**
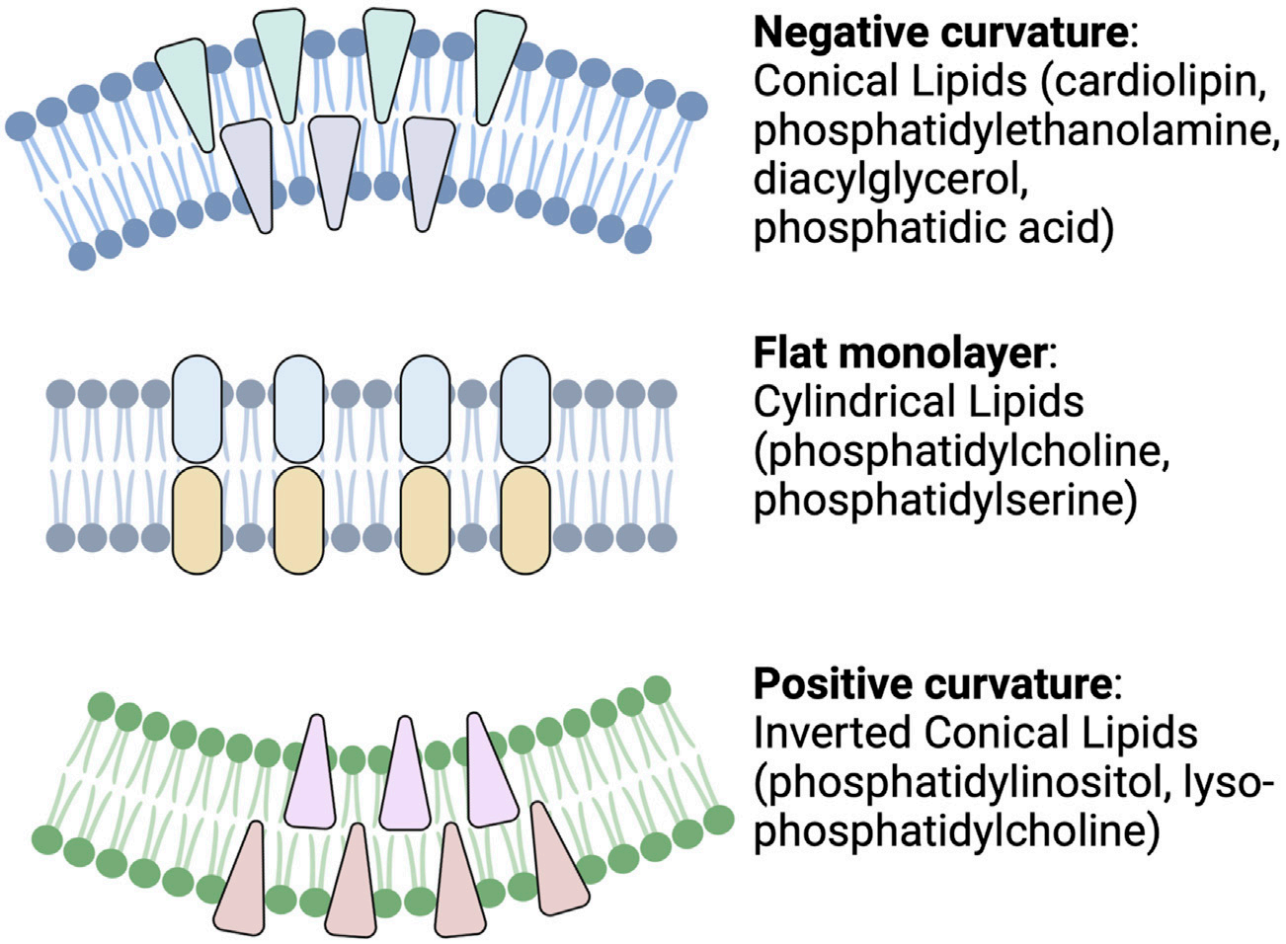
Schematic depicting cellular membrane curvature induced by different lipids. Lipids such as cardiolipin, phosphatidylethanolamine, diacylglycerol, and phosphatidic acid cause negative curvature, while lipids, such as phosphatidylinositol, lysophosphatidylcholine, cause positive curvature of the membrane. Other lipids with cylindrical shapes, such as phosphatidylcholine and phosphatidylserine, do not induce any curvature (Readapted from McMahon and Boucrot, 2015 and created with BioRender.com).

For example, different lipid shapes can result from differences in their headgroups and the length and saturation status of their acyl chains. As shown in Figure 2, cylindrical-shaped lipids, such as phosphatidylcholine and phosphatidylserine, cause the formation of a flat monolayer with no bending. Conical-shaped lipids, such as phosphatidylethanolamine, phosphatic acid, diacylglycerol, or cardiolipin, contain smaller polar head groups, which tend to migrate close to each other and bend the membrane toward the outer space and so cause a negative curvature. Whereas lipids with a large head group, such as lysophosphatidylcholine or phosphatidylinositol phosphates, tend to move away from each other and can cause a bending toward the lumen side and yield a positive curvature. These processes are one mechanism for endocytosis, cargo release, or budding from the membrane of parent cells. Lipid geometry is also affected by the number of double bonds. Generally, these properties may influence exosome function (McMahon & Boucrot, 2015).

As discussed, lipids also influence exosome generation and release from their parent cells. This was illustrated in a study showing that increasing the cellular level of the ether lipid precursor, hexadecylglycerol, increased the ether lipid level in exosomes, which resulted in higher exosome release from PC-3 (adenocarcinoma) cells (Phuyal et al., 2015). Ether lipids (plasmalogens containing arachidonic acid) have also been shown to play a role in the fusion of multivesicular bodies to the plasma membrane (Glaser and Gross, 1994). Lipid domains on exosomal membranes can also serve as sites for some proteins (e.g., Lyn protein kinase of the Src family, flotillin-1, and stomatin) to release into extracellular spaces, showing the effect of lipids on protein sorting in exosomes (de Gassart et al., 2003; Hessvik and Llorente, 2018).

Considering the well-characterized effects of lipid structure on the cell membrane, some concepts might be generalized to the role of lipids in the exosome membrane. In the following section, we introduce some of the main lipid types found in EVs and provide an overview of their role in EV-based biogenesis. Specifically, we discuss the role of four lipid categories: ceramides, cholesterol, phosphatidylserine, and phosphatidic acid.

#### 1.2.1 Ceramides

**FIGURE 3:**
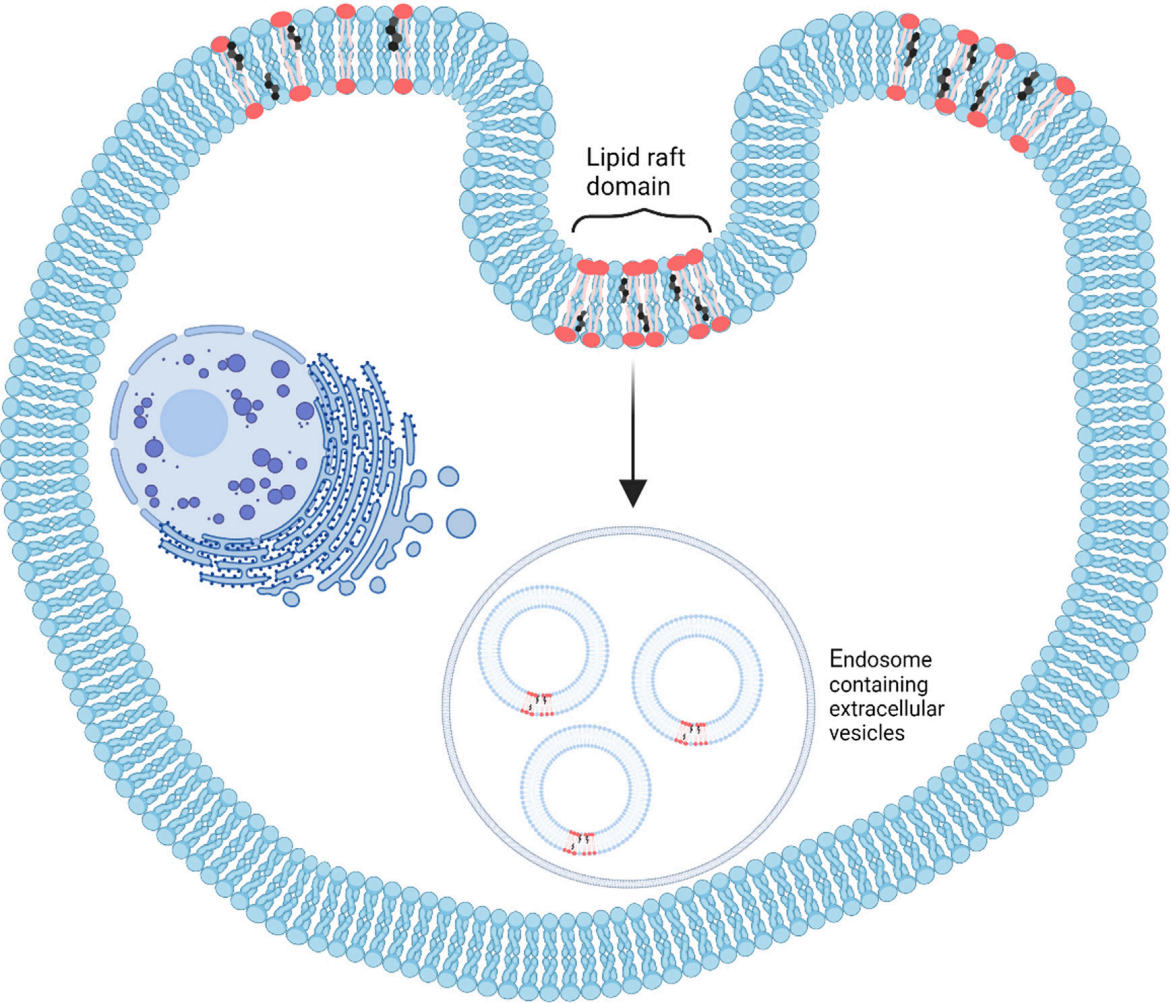
A schematic depicting the formation of lipid rafts, which are microdomains created by dynamic lipid molecules effective in extracellular vesicle biogenesis. These lipid rafts are suitable for binding exosomal components (Readapted from Anderson and Borlak, 2008 and created with BioRender.com).

Ceramides are a type of sphingolipid composed of sphingosine and fatty acids. Two main ceramide species are found in exosomes, C18:0 and C24:1, while C16:0 is more likely found in intracellular vesicles. Increasing C16:0 and C18:0 ceramide concentrations can change the curvature of the cellular membrane leading to a change in vesicle formation and release (Burgert et al., 2017). One way to study the role of ceramides is by first using inhibitors to block neutral sphingomyelinase 2, an enzyme that converts sphingomyelins into ceramides and decreases ceramide levels (Trajkovic et al., 2008). Ceramide generation is an important element for the formation of EVs, independent of the endosomal sorting complexes required for transport (ESCRT)-based pathway involved in exosome biogenesis (Elsherbini & Bieberich, 2018). Both *in vitro* and *in vivo* studies have shown that a reaction catalyzed by neutral sphingomyelinase 2 can lead to changes in exosome secretion (Dinkins et al., 2016). For example, by inhibiting neutral sphingomyelinase 2 in oligodendroglial precursor (Oli-neu) cells, exosome release was decreased (Trajkovic et al., 2008). Ceramides can also destabilize the exosome membrane, leading to cargo release (Elsherbini & Bieberich, 2018), and could be explored to engineer therapeutic delivery vehicles. In addition to negative curvatures induced by ceramide species, cargo sorting into multivesicular endosomes may be influenced by the formation of raft-based microdomains. This phenomenon, depicted in Figure 3, is mediated by sphingolipid-enriched microdomains in the membrane, called “rafts,” which can join to form a large membrane macrodomain by self-association of ceramide molecules through hydrogen binding. This can provide a platform for the binding of exosomal proteins and receptors (Gulbins and Kolesnick, 2003) and a mechanism by which sphingolipids (ceramides) can induce exosomal budding independent of the endosomal sorting complexes required for transport pathway (Trajkovic et al., 2008).

Ceramides may also modulate cell signaling in cancer cells, which could be targeted in potential treatment approaches (de Mera Melero-Fernandez et al., 2021). Although these early studies indicate ceramides' functional and structural role in the formation and function of EVs, there is still much to be discovered concerning understanding their precise role in disease pathogenesis and how these lipid species may be used as a target to influence prognosis.

#### 1.2.2 Cholesterol

Cholesterol is synthesized in the endoplasmic reticulum, mainly in the liver, and is also supplemented by dietary intake. Cholesterol is derived from a precursor called sterane (cyclopentanoperhydrophenanthrene) by enzymes in the cytoplasm, endoplasmic reticulum, and peroxisomes. Although the level of cholesterol in the endoplasmic reticulum is lower than in the plasma membrane, this organelle plays an essential role in regulating cholesterol levels. Other organelles like the endosomal recycling compartment, multivesicular bodies, the trans-Golgi network, and the Golgi apparatus have different cholesterol levels depending on their functionalities. Newly synthesized cholesterol is translocated from the endoplasmic reticulum to the plasma membrane via vesicular or non-vesicular transport. High intracellular levels of cholesterol are toxic for cells since cholesterol molecules cannot be degraded within the cell. Indeed, the cells maintain an equilibrium between cholesterol synthesis and its export or uptake by the lipoproteins (Röhrl and Stangl, 2018; Lee and Bensinger, 2022). Excessive cholesterol levels are restored in lipid droplets after esterification inside the endoplasmic reticulum (Röhrl and Stangl, 2018).

Cholesterol can move quickly in and out of cells by various pathways, and changes in lipid membrane content have been shown to greatly affect cholesterol levels (Maxfield and van Meer, 2010). As in the cell membrane, cholesterol in the exosomal membrane keeps the phospholipid bilayer stable, contributes to structural rigidity, and helps maintain the order of other lipid molecules (Maxfield and van Meer, 2010; Wang et al., 2020). In essence, the hydrocarbon ring structure of cholesterol lends itself to filling empty spaces between lipids. Further, just as the joining of sphingolipid molecules in lipid rafts helped the progression of cargo sorting in EVs, it has been shown that when cholesterol interacts with sphingolipids through hydrogen bonding and hydrophobic interactions, these lipids create discrete membrane structures (Figure 3), which are different from phospholipid arrays in the parent cell membrane (Gulbins and Kolesnick, 2003). High levels of cholesterol in the membrane can act as a barrier and decrease the permeability of small molecules passing through the membrane (Lippincott-Schwartz and Phair, 2010), which is relevant from an EV stability/engineering perspective.

Studies have shown that decreasing cholesterol, ceramide, and phosphatidylcholine levels in microvesicular bodies leads to a decrease in exosome release with the help of several lipidomic enzymes (Hessvik and Llorente, 2018; Abdullah et al., 2021). These enzymes can also activate phosphatidylcholine, which then leads to the formation of intraluminal vesicles. For example, western blotting of exosome markers in apolipoprotein E-deficient mice astrocytes showed that exosome release was decreased when the cellular level of cholesterol increased. This could be mediated by the phosphoinositide-3-kinase–protein kinase B/Akt pathway, one possible pathway for exosome release (Hessvik and Llorente, 2018; Abdullah et al., 2021). Cholesterol also plays a role in the cellular uptake of exosomes. For example, exosomes released by Mantle cell lymphoma cells were shown to be taken up by B-lymphocytes through proteins associated with cholesterol/lipid raft microdomains (Wang et al., 2020).

Cholesterol can help load vesicles in late endosomes into multivesicular bodies, which might be mediated by protein transporters, such as ATP binding cassette transporter subfamily G. Indeed, it was shown that only cultured B lymphocytes enriched in cholesterol could fuse with the cell membrane to release exosomes (Pfrieger and Vitale, 2018). Another mechanism regulated by cholesterol is the assembly of the ESCRT system. While these complexes do not bind directly to cholesterol, they bind to phosphoinositides, which leads to the formation of clusters with cholesterol (Boura et al., 2012). These interactions then activate ESCRT-based pathways, cargo sorting, and in general, EV biogenesis.

Concerning influences on disease states, the accumulation of lipids, such as cholesterol and cholesterol esters, has been confirmed in the brain of individuals with Alzheimer’s disease (Loera-Valencia et al., 2019; Bok et al., 2021). This, in turn, has been associated with the induction of tau pathologies and characteristic neurofibrillary tangles associated with memory loss and dementia. High cholesterol levels can also lead to other Alzheimer’s disease characteristics, such as vesicular trafficking defects and increases in amyloid-beta secretion (Bok et al., 2021). Further work is needed to characterize the role of cholesterol concentrations and movement within the brain via EVs, perhaps via tracking in apolipoprotein E-deficient animal models.

#### 1.2.3 Phosphatidylserine

Phosphatidylserine is an abundant lipid that typically exists at the inner leaflet of the cell membrane. However, it can become exposed at the outer leaflet of the cell membrane in tumor cells (Leventis & Grinstein, 2010; Skotland et al., 2019). Phosphatidylserine is critical for enzyme activation and apoptotic cell clearance, while its negative charge enables the cell membrane to interact with cationic molecules and proteins (Leventis and Grinstein, 2010). Phosphatidylserine within the EV membrane is also considered an important constituent, given these variable roles. For example, EVs have been shown to impact coagulation, platelet aggregation, and thrombosis, which was mediated by negatively charged phosphatidylserine molecules (Ståhl et al., 2019). In addition, recent evidence has suggested that phosphatidylserine ‘introduces’ exosomes to macrophages. Specifically, negatively charged phosphatidylserine, exposed on the surface of the intravenously injected B16BL6-derived exosomes, increased exosome uptake by liver macrophages (Matsumoto et al., 2017). Lastly, it has been shown that exosomes can bind to immunomodulatory receptors through phosphatidylserine molecules, which likely mediates entry into recipient cells (Miyanishi et al., 2007). Taken together, this evidence suggests that phosphatidylserine can be selectively used to modulate uptake by cells, which may be utilized in drug delivery or diagnostic applications and become particularly relevant in immune and cancer-targeting applications.

#### 1.2.4 Phosphatidic acid

Phosphatidic acid is a cone-shaped phospholipid produced by the hydrolysis of phosphatidylcholine catalyzed by the enzyme, phospholipase D (Borel et al., 2020). Phosphatidic acid contains a small headgroup, which can cause a negative membrane curvature (Egea-Jimenez and Zimmermann, 2018; Tanguy et al., 2019). Phosphatidic acid can affect both the number of EV produced/released form cells and the EV composition. Specifically, phosphatidic acid helps the formation of intraluminal vesicles by directly binding to proteins, such as annexins and syntenin, through the budding of intraluminal vesicles, which induces release (Egea-Jimenez and Zimmermann, 2018). Phosphatidic acid affects exosome composition by interacting with signaling proteins, such as proto-oncogene tyrosine-protein kinase Src, or cytosolic proteins, such as protein kinase η_₀_ and helps anchor them onto exosomes (Egea-Jimenez and Zimmermann, 2018). M. Borel *et al.* uncovered a possible role of phospholipase D in exosomes released from phospholipase D-overexpressed human prostate cancer cells in inducing proliferation and differentiation of clonal murine cells of immature osteoblasts (MC3T3-E1 cells) (Borel et al., 2020). Phospholipase D can also induce proliferation by activating both the MAPK and the PI3K/Akt pathways, which are responsible for cell proliferation (Bruntz et al., 2014). Exosomes released from C4-2B cells treated with a phospholipase inhibitor could not stimulate proliferation or differentiation of MC3T3-E1 cells and could not activate *in vitro* calcium deposition in MC3T3-E1 cells (Borel et al., 2020). In addition, exosome secretion from C4-2B cells decreased by inhibiting phospholipase D. By combining phosphatidic acid with a phospholipase D inhibitor, exosome secretion increased, which substantiated the role of phosphatidic acid in exosome secretion from C4-2B cells (Borel et al., 2020). Taking inspiration from the role of phosphatidic acid in cell membranes, it may be used in engineering EVs to investigate possible mechanisms to increase EV production.

Other lipids have been shown to affect the biogenesis, function, and fate of different vesicles. Critically, the lipid composition of the plasma membrane can affect the performance and formation of vesicles that pass through the blood-brain barrier. For example, docosahexaenoic acid, a long-chain omega-3 polyunsaturated fatty acid, can inhibit the transport of vesicles through the blood-brain barrier and result in a brain that is less susceptible to toxins. Accordingly, decreased levels of docosahexaenoic acid may lead to a ‘leaky’ blood-brain barrier, which can contribute to neurodegenerative disease development (Chew et al., 2020). Docosahexaenoic acid can also change the cargo loading of exosomes. In one study, exosomes derived from breast cancer cells treated with docosahexaenoic acid showed an increase in their small RNA content (Hannafon et al., 2015). Considering this feature of docosahexaenoic acid, engineering exosomes using it in their membrane might be helpful in cargo loading for therapeutic delivery.

It is important to emphasize that apart from the individual role of diverse lipid components and the enrichment of each lipid in the exosome membrane, the distribution ratio of different lipids may be a key factor in determining fate and function. Indeed, a common narrative in the exosome literature is the differences between the lipid composition of parent cell membranes and their exosomes (Skotland et al., 2019). For example, lipidomic analysis showed that the enrichment ratio of ceramides in exosomes is about 1.3 to 3 fold more than in the membrane of parent cells, and its ratio is three folds more than cholesterol (Elsherbini and Bieberich, 2018). These ratios are equally important to elucidate when studying EV mechanisms and in engineering for biomedical applications.

In other words, while the roles of lipids are not as well characterized after their release from cells, it is evident that we should consider that any changes in lipid type may change the total distribution of lipids in the EV membrane, which will ultimately affect their performance, fate, and mechanism of action. As a result, more detailed compositional data and comprehensive lipidome studies are needed to characterize complex dynamics at a physiological level, which can then inform the synthesis and engineering of exosomes for therapeutic applications. This way, we can make more organized and “smarter” nanovesicles suitable for treating various diseases or even to influence distribution, uptake, and metabolism in cells. Even biomarker studies could benefit from enhancing their data pools to include the EV lipidome to use as new molecular targets for disease diagnostics (Skotland et al., 2017).

## 2 The extracellular vesicle and lipid biogenesis

Various lipids, whether in the cell membrane or EVs themselves, affect their biogenesis. From this perspective, it is important to understand lipid sources and how cells regulate different lipids for the cell membrane or EV membrane. In this section, we introduce the concept of EV formation and lipid sources, which form intricate arrays in the membrane.

**FIGURE 4:**
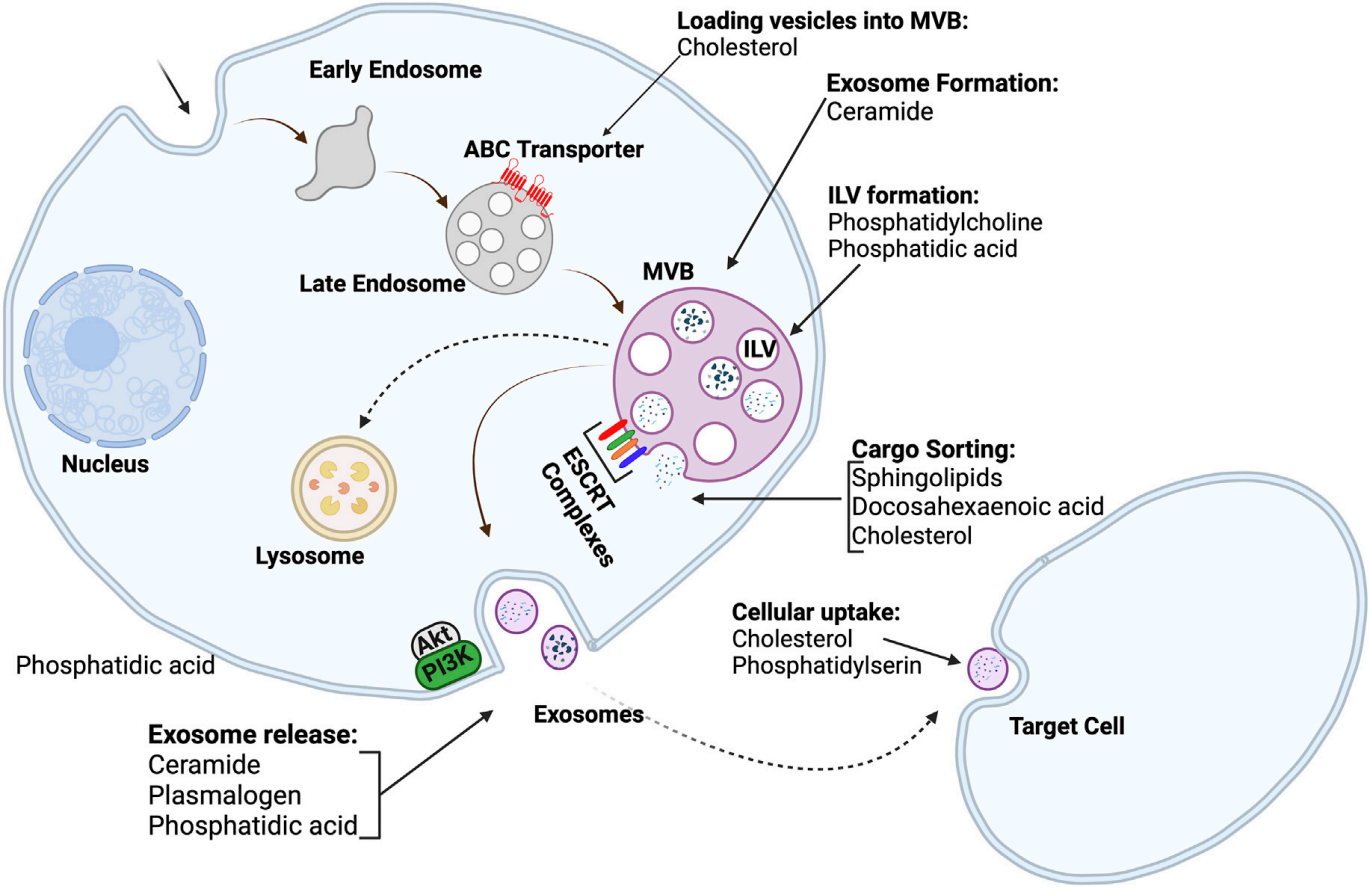
Exosome biogenesis and lipid roles in different stages of exosome biogenesis; Intraluminal vesicles are produced by the budding of the cell membrane into the lumen of endosomes. The endosomes then transform into multivesicular bodies. Multivesicular bodies can either be directed to lysosomes to remove cellular debris, or they can fuse with the cell membrane to release exosomes into extracellular space. The roles of many lipids in different stages of exosome biogenesis are also depicted (Created with BioRender.com).

Exosome biogenesis, in particular, is a process in which the endosomal membrane is invaginated to form early endosomes. Early endosomes mature to form late endosomes, which finally transform into multivesicular bodies. As shown in Figure 4, multivesicular bodies contain intraluminal bodies, which can merge either with lysosomes to degrade as cellular waste or with the plasma membrane to release the intraluminal vesicles as exosomes (Simons & Raposo, 2009; Mathivanan et al., 2010). The mechanism for the formation of intraluminal vesicles and multivesicular bodies is the previously described ESCRT machinery, which consists of four complexes and related proteins, including vacuolar protein aborting-associated protein 4 and ALG-2-interacting protein X (Egea-Jimenez & Zimmermann, 2018).

**FIGURE 5:**
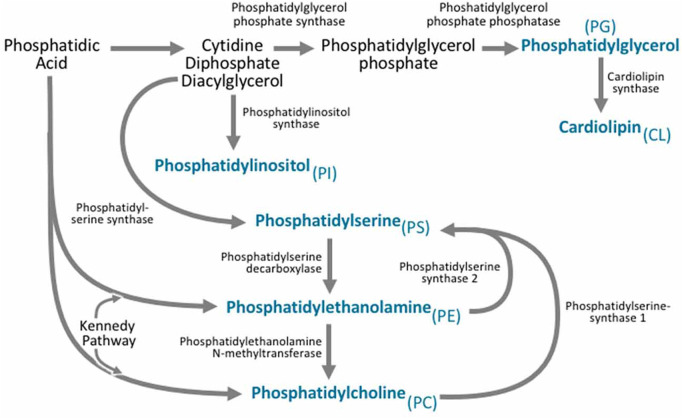
Phospholipid Biogenesis. Phosphatidic acid is involved in phospholipid biogenesis through two different synthetic pathways. Phosphatidylethanolamine is synthesized through two pathways: The cytidine diphosphate-ethanolamine pathway and the phosphatidylserine decarboxylation pathway and it can also be derived from phosphatidylserine. Phosphatidylcholine can be produced through the Kennedy pathway and also the enzyme phosphatidylethanolamine N-methyltransferase can use phosphatidylethanolamine as a precursor to produce phosphatidylcholine. Phosphatidylserine is produced from both phosphatidylcholine and phosphatidylethanolamine (Reproduced from Tracey et al., 2018).

Organelles synthesize cellular lipids termed “lipid droplets” (Pol et al., 2014; Wilfling et al., 2014), which store and supply lipids for many cell activities, such as energy metabolism and membrane synthesis. Lipid droplets can be produced by increases in neutral lipids in the endoplasmic reticulum. Many enzymes and metabolic reactions are involved in synthesizing lipid droplets, including acyl-CoA synthetases for neutral lipids production, fatty acid activation by coenzyme A, and phospholipid synthesis (Pol et al., 2014). During lipid droplet biogenesis, the phospholipid content within the lipid droplets also increases. Phospholipid synthesis (Figure 5) can occur via two pathways, the Kennedy pathway, which is a *de novo* phospholipid synthesis, and the Lands cycle. The communication between lipid droplets and the endoplasmic reticulum is critical for lipid droplet biogenesis since many biochemical reactions and enzymes involved in lipid droplet biogenesis are active in the endoplasmic reticulum (Pol et al., 2014). In addition to the role of the endoplasmic reticulum, other organelles like the mitochondria and peroxisomes are involved. For example, the synthesis of ether-linked phospholipids only occurs in peroxisomes (Pol et al., 2014).

Understanding the reaction and enzymes involved in the biosynthesis of different lipids in cells can help researchers identify which lipids should be intentionally manipulated to improve exosome production through cell culture, genetic engineering, or surface modifications. As EV lipids are derived from cellular lipid sources, the lipid biogenesis of EVs is not different from their parental cells, but based on published investigations, there is limited data on the mechanisms involved in the assembly of lipids either in the cell membrane or in the EV membrane. More research could help modulate EV behavior by manipulating the lipid assembly and content.

### 2.1 Pathological changes in lipid composition of extracellular vesicle membranes

Recent work has shown that changes from normal cell conditions to a pathophysiological state can alter the lipid array of EV membranes. This could be used as a practical tool for biomarker purposes to detect diseases by assessing exosomal lipids or as a route of intervention. For example, in one study, the lipid composition of extracellular exosomes derived from the frontal cortex tissue of patients with Alzheimer’s disease was assessed using semi-quantitative mass spectrometric analysis. The results indicated that the concentration of glycerophospholipids, diacyl-glycerophosphoethanolamine, and polyunsaturated fatty acid-containing phosphatidylethanolamine molecules decreased, while sphingolipids and plasmalogen lipid, increased compared to controls (Su et al., 2021). In another study, microglial EVs isolated from the cerebral cortex of individuals with Alzheimer’s disease showed an increase in free cholesterol, upregulation of bis(monoacylglycerol) phosphate (BMP 36:2), and mono-hexosyl ceramides (mhCerd18:1/24:1) and a decrease in phosphatidylethanolamine 38:0 and 38:1. The study also showed a significant increase in phosphatidic acid 40:6, phosphatidylserine 40:6, and docosahexaenoic acid-containing polyunsaturated lipids (Cohn et al., 2021). These changes in lipids are important for EV formation and release and could affect EVs role in intracellular trafficking, and may contribute to the progression of Alzheimer’s disease.

Changes in EV lipids in pathological states are not limited to Alzheimer’s disease. These phenomena were also confirmed in exosomes derived from cancer cells, such as poorly metastatic melanoma cells and highly metastatic melanoma cells. Using matrix-assisted laser desorption/ionization-time of flight mass spectrometry, data showed a decrease in phosphatidylcholine, phosphatidylethanolamine, phosphatidylserine, and plasmenylcholine species and an increase in lysophosphatidylcholine in exosomes of both poorly and highly metastatic melanoma cells. Their exosome lipid composition showed an increase in sphingomyelins, glycerophospholipids (phosphatidic acid, lysophosphatidylcholine), and phospholipid bis (monoacylglycero) phosphate. There were also high levels of polyunsaturated species of phosphatidylcholine and plasmenylcholine, which promotes efficient uptake by neighboring recipient cells (Lobasso et al., 2021). In another example, the lipidomic analysis of exosomes derived from the LIM1215 colorectal cancer cells using matrix-assisted laser desorption ionization-time-of flight/mass spectrometry confirmed an increase in sphingolipids, sterol lipids, glycerolipids, and glycerophospholipids, and particularly plasmalogen- and alkyl ether-containing glycerophospholipids (Lydic et al., 2015). It should be noted that these disbalances in EV lipids might affect their performance, destination, and release. All these assumptions need to be investigated further to understand why the cellular response to certain pathological conditions is reflected in the exosome lipidome and how these changes can help cells survive.

Changes in EV lipids have also been observed in lipid subclasses, especially ceramide species, which seem to have an important role in cancer. One study analyzed exosomes derived from human bronchial epithelial (HBEC-12KT) and mesenchymal-like prostate carcinoma cells using comparative high-performance liquid chromatography-tandem mass spectrometry. Results showed similar levels of sphingolipids, glycosphingolipids, and decreased levels of sphingosine and lactosylceramide in exosomes of transformed cells compared to exosomes from non-transformed cells (Machala et al., 2021). However, the levels of both sphingosine and lactosylceramide were higher in the transformed cells. The lipid composition in exosomes derived from different *in vitro* glioblastoma brain tumor subtypes, such as mesenchymal cells with semi-nodular or nodular dissemination and proneural disseminations, were assessed using ultra-high-performance liquid chromatography-quadrupole time-of-flight mass spectrometry. Although C18 ceramide species were the most common ceramide in the brain, C16 ceramide and C24:1 ceramide were the highest ceramide species in both glioblastoma cells and their derived exosomes. A dramatic increase in the C24:1 ceramide in mesenchymal cells with semi-nodular disseminations was found. Also, an increase in C16 ceramide and a decrease in C24:1 ceramide in proneural disseminations were observed in exosomes compared to their parent cells (de Mera Melero-Fernandez et al., 2021).

EV lipid imbalances could be reflected in biological samples, such as urine or blood, which may be a good disease indicator and significant for clinical diagnostics. For example, comparing urinary exosomes from individuals with prostate cancer with exosomes derived from PC-3 cells confirmed that urinary exosomes have higher cholesterol and lower phospholipid concentrations compared to PC-3 cell-derived exosomes (Skotland et al., 2017). In contrast, both have similar sphingomyelin levels. Urinary exosomes have higher phosphatidylserine, while exosomes from PC-3 cells have higher phosphatidylcholine concentrations. In addition, phosphatidylethanolamine was only detected in urinary exosomes. The level of hexosylceramide and lactosylceramide species increased in urinary exosomes compared to healthy controls. In addition, both urinary exosomes and PC-3 cells had higher levels of cholesterol and sphingomyelin. For clinical purposes, Sharma et al. (2017). could detect cancer in blood samples by considering disbalances in exosome lipids. They developed an enzyme-linked immunoassay technique to detect phosphatidylserine-enriched exosomes in the blood of cancer patients with ovarian malignancies and determined that exosomes containing phosphatidylserine on their surface were significantly higher compared to those isolated from control cases. This work is consistent with previous evidence indicating that phosphatidylserine molecules were overexpressed on the surface of tumor cell membranes, which were additionally released as plasma exosomes (Sharma et al., 2017; Matsumura et al., 2019).

Table 1 summarizes changes that have been observed in the EV lipidome in atypical cells or pathological states, which may be relevant for diagnostic and intervention-development purposes. By gathering more information on lipid changes of the EV membrane in different cells/conditions, data can be used to create patterns or lipidomic maps to help diagnose or monitor various diseases.

**TABLE 1:** Changes in the lipid composition of EVs derived from different disease states.

EV source	Method (Lipid analysis)	Increased in EV membrane lipids	Decreased in EV membrane lipids	Reference
Alzheimer’s disease frontal cortex tissue	Semi-quantitative mass spectrometric	-Sphingolipids	-Glycerophospholipids	Su et al. (2021)
		-Plasmalogen lipid	-Diacyl-phosphatidylethanolamine	
			-Polyunsaturated fatty acids containing phosphatidylethanolamine	
Alzheimer’s disease cultured cortical neurons (In vivo)	Liquid chromatography–mass spectrometry	-Ceramide		Su et al. (2021)
		-Dihydroceramide		
		-Dihydrosphingomyelin		
Microglial	Liquid chromatography tandem mass spectrometry	-Free cholesterol	-Phosphatidylethanolamine 38:0 & 38:1	Cohn et al. (2021)
		-Bis(monoacylglycero) phosphate	-Phosphatidic acid 40:6	
		-Mono-hexosyl ceramides	-Phosphatidylserine 40:6	
			-Docosahexaenoic acid-containing polyunsaturated lipids	
Murine neuroblastoma N2a cell line (In vitro)	Liquid chromatography–mass spectrometry	-Sphingolipids (Mono-hexosyl ceramides & Lactosylceramide)		Miranda et al. (2018)
		Phospholipid		
		-Bis(monoacylglycero) phosphate		
LCP and SK-Mel28 cell lines	Matrix-assisted laser desorption ionization-time-offlight/mass spectrometry	-Lysophosphatidylcholine	-Phosphatidylcholine	Lobasso et al. (2021)
		-Sphingomyelin	-Phosphatidylethanolamine	
		-Glycerophospholipid species (phosphatidic acid)	-Phosphatidylserine	
		Bis(monoacylglycero)phosphate	-Plasmenylcholine species	
		Polyunsaturated species of phosphatidylcholine		
		-Polyunsaturated plasmenylcholine		
LIM1215 colorectal cancer cells	Matrix-assisted laser desorption ionization-time-offlight/mass spectrometry	-Sphingolipids		Lydic et al. (2015)
		-Sterol lipids		
		-Glycerolipids		
		-Glycerophospholipids		
		-Plasmalogen- & alkyl ether-containing glycerophospholipids		
Urinary exosomes of prostate cancer patients	Hybrid triple quadrupole/linear ion trap mass spectrometer	-Hexosylceramide species		Skotland et al. (2017)
		-Lactosylceramide species		
		-Sphingomyelin		
		-Cholesterol		
		-Phosphatidylethanolamine		
PC-3 cell line	Hybrid triple quadrupole/linear ion trap mass spectrometer	-Sphingomyelin		Skotland et al. (2017)
		-Cholesterol		
		-Phosphatidylcholine		
Comparing transformed mesenchymal HBEC-12KT-B1 cells with non-transformed parental epithelial cells	Comparative high-performance liquid chromatography-mass spectrometry/mass spectrometry		-Sphingosine	Machala et al. (2021)
HBEC-12KT cells			-Lactosylceramide	
Glioblastoma brain tumors	Ultra-high-performance liquid chromatography-quadrupole time-of-flight mass spectrometry	-C24:1 ceramide (in MES-SN phenotype)	-C24:1 ceramide (in PN phenotype)	de Mera Melero-Fernandez et al. (2021)
		-C16 ceramide (in PN phenotype)		
Blood of patients with ovarian malignancies	Using gold nanoparticles	-Phosphatidylserine		Matsumura et al. (2019)

### 2.2 Lipid metabolism involvement and extracellular vesicle lipid cargo in Alzheimer’s disease

Many factors, such as an unhealthy lifestyle or genetic history, can cause disruptions in lipid metabolism. For example, several genes are involved in lipid metabolism and Alzheimer’s disease, including the apolipoprotein epsilon4 allele, which is a major genetic risk factor for Alzheimer’s disease (Chew et al., 2020). It has even been suggested that unbalanced lipid levels may cause Alzheimer’s disease (Miranda et al., 2018; Czubowicz et al., 2019). For example, phosphatidylinositol-3-phosphate reduction was observed in individuals diagnosed with Alzheimer’s disease. Researchers studied the effect of suppressing a kinase (Class III phosphoinositide 3-kinase), which led to autophagy inhibition that induces the release of exosomes containing lipids such as phospholipid bis (monoacylglycero) phosphate or lysobisphosphatidic acid and amyloid precursor protein fragments from N2a cells. In other words, as an alternative response, these new exosomes can eliminate cell debris that was not degraded normally by endolysosomal function. Lipidic analysis of these new exosomes showed an increase in sphingolipids (monohexosylceramide and lactosylceramide) and phospholipid bis(monoacylglycero) phosphate. The class III PI 3-kinase Vps34 inhibition also affected lipid metabolism in cultured cortical neurons with a dramatic increase in ceramides, dihydroceramide, and dihydrosphingomyelin. Phospholipid bis (monoacylglycero) phosphate levels also increased in patients with Alzheimer’s disease. This abnormal accumulation of sphingolipids can lead to the inhibition of lysosomal enzymes and sensitizing the endosomal membrane. Ultimately, this imbalance may accelerate the progression of neurodegenerative disease (Miranda et al., 2018). Increased ceramide levels (Czubowicz et al., 2019) and exosome biogenesis (Miranda et al., 2018) are some other metabolic changes seen in Alzheimer’s disease.

Evidence has also shown that exosomes derived from other cell types could affect lipid metabolism under pathophysiological conditions. For example, under hypertrophic conditions, exosomes derived from C2C12 skeletal muscle cells can induce lipolysis in adipose tissue. In addition, they observed a significant increase in ceramide species (C16, C18:1, C24:0 and C24:1) and diacylglycerol containing unsaturated fatty acids, such as palmitoyl-arachidonoyl, but a reduction in C20 and C22 species (Valentino et al., 2021). Exosomes can affect lipid metabolism in adipose tissue since they can transport enzymes, such as phospholipases A2, cytosolic phospholipases A2, calcium-independent phospholipases A2, phospholipase D2, diglyceride kinase, phosphoinositide phosphatase related to lipid metabolism (Record et al., 2014). Thus, these enzyme carriers might be triggered to modulate lipid metabolism in a way other than altering the genes of relevant enzymes.

### 2.3 Interactions between extracellular vesicle lipid and cytotoxic proteins in neurodegenerative disease

Based on previous results demonstrating the affinity of lysine- and arginine-rich peptides to phospholipid bilayers (Eguchi et al., 2001; Gratton et al., 2003), there is an equally good affinity between lysine residues in peptides and phospholipids in exosome membranes. Interestingly, an exosome isolation technique has even been designed to isolate exosomes based on ‘lipid-binding peptides’ (inspired by this affinity), which has applications even beyond the cancer cell model described (Ishida et al., 2020). In this section, we introduce studies related to interactions between EVs and cytotoxic proteins, namely, tau and amyloid β, to draw attention to the potential role of exosomal lipids in neurodegenerative disease and the development of new targets and/or diagnostic approaches.

The interaction of tau, a protein involved in the progression of Alzheimer’s disease, with exosomal membranes is poorly understood. However, by investigating the interaction of tau with negatively charged vesicles, the formation of tau-phospholipid complexes with high stability and their toxicity to the primary hippocampal cultures was confirmed. Tau-phospholipid complexes showed more neuronal uptake compared to monomeric tau (Ait-Bouziad et al., 2017). Tau molecules also bound to synaptic vesicles via their N terminal domain. Pathologic tau can increase the level of F-actin molecules in presynaptic terminals, leading to decreased synaptic vesicle mobility (Zhou et al., 2017). These studies revealed the role of exosomes in tau pathology and may be used to develop new ways of regulating tau toxicity in brain cells to attenuate the progression of Alzheimer’s disease.

**FIGURE 6:**
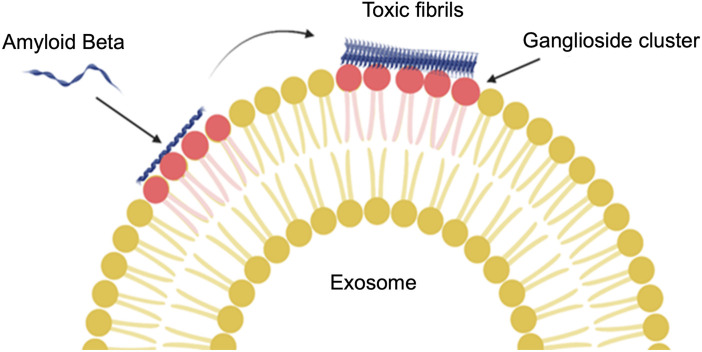
The interaction of glycosphingolipids on an exosome membrane with amyloid β. Ganglioside (a type of glycosphingolipid) molecules join together and then amyloid β can bind to the exosomes. The conformation change from α-helix to β-sheet will be reflected in the amyloid β structure by binding to the ganglioside cluster (Created with BioRender.com).

β-secretase is an enzyme that cleaves the amyloid precursor protein to amyloid β peptides in early endosomes, which can ultimately lead to an increase in amyloid β plaques. Research has shown that both β-secretase and amyloid precursor protein (APP) interact with cell membrane lipids (cholesterol, sphingolipids, and gangliosides) (Simakova and Arispe, 2007; Chew et al., 2020). Indeed, though the relationship between cytotoxic proteins, such as amyloid β peptide and tau protein, and lipids is not entirely clear, the movement of exosomes and apoptosis activation is an important factor to consider in this context. For example, the inhibition of ceramide production prevented apoptosis induced by amyloid β in astrocytes. C18 ceramides increased in astrocytes exposed to the amyloid peptide, which was important for the secretion of exosomes induced by amyloid peptides (Wang et al., 2012). The relationship between ceramide and amyloid β was also confirmed in recent research, where it was found that exosomes enriched in ceramides not only carry amyloid β but also make N2a cells sensitive to the toxicity of amyloid β with a mechanism different from plaque formation. They found that ceramides are important for the interaction of amyloid β with exosomes (Elsherbini et al., 2020). It has also been shown that glycosphingolipids on exosome membranes (Figure 6) are necessary for the binding of amyloid β to exosomes derived from neuroblastoma cells both *in vivo* and *in vitro* (Yuyama et al., 2014). Ganglioside molecules make clusters on the exosome membrane that to bind amyloid β (Matsuzaki et al., 2010).

Overall, EV lipids appear to interact with cytotoxic proteins involved in the progression of Alzheimer’s disease. It might be possible to block the lipid interaction sites to prevent the potential cargo loading of cytotoxic proteins in EVs to reduce their spread throughout the brain or load EVs with lysosomal enzymes to destroy cytotoxic proteins. Lipids may be more important than expected, though more research is needed to determine the role of lipids in treating Alzheimer’s disease and other neurodegenerative diseases.

## 3 Analytical techniques for lipid isolation and analysis

An integral component of EV-based lipid work is effective characterization and analysis. Lipid extraction techniques vary depending on the sample source, type, and the intended use of the extracted lipids. While the process of isolating lipids from biological samples can be challenging due to their chemical and structural diversity. Generally, three factors should be considered when isolating lipids from a biological sample: the type and volume of the sample, the lipids of interest, and the intended purpose of the isolated lipids, such as downstream analysis or physical characterization (Furse et al., 2015). In the context of EV analysis, an additional consideration is the potential heterogeneity of the sample and effective enrichment or fractionation. For example, the smaller abundance of lipids or yield in a sample may require special extraction considerations or advanced techniques, such as supercritical fluid chromatography fast-scanning triple quadrupole mass spectrometry (SFC/QqQMS) (Nishida-Aoki et al., 2020). While contamination with lipid droplets or lipoparticles should also be considered and depends on the isolation technique (Skotland et al., 2017). Herein, we provide a brief discussion of lipid extraction techniques and analysis.

Effective lipid extraction necessitates the consideration of factors such as extraction efficiency and complete removal of non-lipid components. Therefore, selecting a suitable solvent that can dissolve both non-polar and polar lipids is crucial. The ‘classic’ lipid extraction methods introduced by Folch et al. (Folch et al., 1957) and later developed by Bligh and Dyer (Bligh & Dyer, 1959) are still widely used. The Folch method is more suitable for solid tissues, while the Bligh and Dyer method works well for biological fluids. In a review by Young-Soo Keum et al., the importance of selecting the appropriate extraction solvent is emphasized, as the properties of the solvent can affect both lipid extraction efficiency and the overall efficacy of the method (Saini et al., 2021). The review lists various solvents recommended for different sample types. For instance, the best solvent mixture for lipid extraction from human plasma samples is shown to be 1-butanol/methanol (1:1, v/v), while methanol/methyl-tert-butyl ether is suitable for sphingolipidomic studies (Saini et al., 2021). However, these classic extraction methods have limitations when it comes to evaluating large sample volumes, and they tend to be time-consuming and labor-intensive. Recently, other advanced techniques, such as microwave-assisted extraction, supercritical fluid extraction (or supercritical CO_2_ extraction), and ultrasonic-assisted extraction, have overcome some limitations of these classic methods by decreasing the degree of solvent needed and decreasing labor time. Still, these techniques can be expensive and unsuitable for evaluating low amounts of fatty acids, which is particularly relevant in EV research. Notably, supercritical CO_2_ extraction, which allows for the selective extraction of lipids and other metabolites, does not require the use of solvents, making it environmentally friendly. However, it can be costly and less effective for extracting polar lipids (Saini et al., 2021).

In terms of lipid identification, gas chromatography is a well-established method for various sample types, providing detailed quantitative analysis when paired with mass spectrometry or flame ionization detection (Furse et al., 2015; Harrison & Watts, 2022). High-performance liquid chromatography (HPLC) is another valuable technique that allows for the isolation of specific components on a smaller scale, particularly suitable for analyzing fatty acids with unstable functional groups (McDonald et al., 2007; Degano & La Nasa, 2017; Donno et al., 2020; Harrison & Watts, 2022). Mass spectrometry is generally considered the gold-standard analysis techniques, offering well-developed sample and data processing and comprehensive insights into the lipid components and fatty acid analysis (Sullards et al., 2011; Bai et al., 2023; Gowda et al., 2023; Hu et al., 2023; Lewis et al., 2023). Wang et al. highlighted significant advancements in lipidomic analysis achieved through the development of various mass spectrometry methods, enabling easier and more accurate lipid analysis (Wang et al., 2015). One example is matrix-assisted laser desorption/ionization mass spectrometry (MALDI-MS), a powerful tool for high-accuracy imaging with excellent spatial resolution, particularly beneficial for *in vivo* lipid analysis. Another notable technique is time-of-flight secondary ion mass spectrometry (TOF-SIMS), which utilizes gold and silver ions to determine cellular lipid contents in EVs and tissues (Wang et al., 2015). Atmospheric pressure chemical ionization mass spectrometry (APCI-MS) is capable of ionizing various lipids, including protonated molecular ions of phospholipids, free fatty acids, and sterols. On the other hand, atmospheric pressure photoionization mass spectrometry (APPI-MS) can be utilized to analyze neutral lipids and phospholipids with higher sensitivity and lower detection limits compared to atmospheric pressure chemical ionization mass spectrometry. However, it is important to note that atmospheric pressure chemical ionization mass spectrometry is less gentle compared to other ionization techniques, such as electrospray ionization mass spectrometry (ESI-MS). ESI-MS focuses primarily on (quasi) molecular ions of lipids and selectively ionizes lipid molecules based on their charges. This selective ionization capability allows for the analysis of different lipid classes without the need for chromatography, providing higher efficiency and sensitivity (Wang et al., 2015). These advancements in mass spectrometry have greatly contributed to the field of lipid analysis, allowing researchers to obtain detailed insights into lipid composition and distribution with enhanced precision and efficiency.

Furthermore, there are notable studies that focus on specific areas of lipidomic research related to membrane bending stiffness, viscosity, and of membrane-bound vesicles, and density. For instance, Steinkühler et al. conducted lipidomic studies to probe the bending rigidity of the plasma membrane in large vesicles (Steinkühler et al., 2019). The measurements were conducted in this investigation using fluctuation analysis, which uses phase contrast images to probe membrane fluctuations (Monzel and Sengupta, 2016). It is unclear whether these analyses could be translated to smaller EV studies, as these large vesicles were on the order of microns; however, this approach could provide insides into the effect of lipid composition on the complex dynamics and potential function relating to EV membrane stiffness and the presentation of receptors. Jeong et al. explored lipidic studies in EVs derived from glioblastoma cells (Jeong et al., 2023). These studies exclusively utilized lipidomic approaches for clinical applications, highlighting the significance of lipid analysis in gaining a deeper understanding of these intriguing biomolecules. Such studies contribute to the growing attention and interest in conducting further lipid analysis to enhance our knowledge in this field.

Generally, to enhance the prominence of lipidomic studies, alongside proteomics and nucleic acid studies, there is a pressing need to develop a cost-effective and efficient lipid analysis technique. This would contribute to unraveling the unknown aspects of lipidomics while minimizing the labor-intensive nature of the current methods. To make EV-focused lipidomic studies more prevalent alongside proteomics and nucleic acid studies, it is crucial to focus on improving the speed, affordability, and labor efficiency of lipid analysis techniques. By streamlining the process, researchers can save time, facilitate larger-scale investigations, and enhance the identification and quantification of lipids. Reducing costs associated with lipid analysis is also important for wider implementation and increased interest. Developing automated or semi-automated approaches, specifically with respect to coordination with EV isolation methods and enrichment can alleviate the labor-intensive nature of current techniques, freeing up researchers to explore the intricate relationships between lipids, proteins, and nucleic acids. This integration will lead to a more comprehensive understanding of biological systems and expand our current understanding of how lipids contribute to EV activity.

## 4 Engineering extracellular vesicles; altering lipid composition

Given the potential for EV use in medical applications, including monitoring disease progression (Peinado et al., 2012), as drug delivery carriers (Vader et al., 2014), vaccination tools (Viaud et al., 2010), and cell therapy (Rani et al., 2015), recent attempts have been made to address challenges, such as cargo loading, purification and isolation, and non-specific targeting (Yang & Wu, 2018; Li et al., 2019). In addition, lipids and proteins on the membrane of EVs can be tailored to influence physiological behavior (Stremersch et al., 2016). Thus, by monitoring and changing the lipid components of EVs, new designs could be promising. Two general strategies have been used to engineer the lipidomic aspect of EV membranes: a hybrid liposome and bioinspired approach and decorating EVs using lipid moieties.

### 4.1 Decorating natural extracellular vesicles

One approach to functionalizing EVs is using lipid moieties as a linker or anchor on the surface of the exosome membrane. As lipids are not genetically encoded like proteins, they must be introduced to the exosome membrane by physical and chemical reactions. For example, in one study lipids were used as a linker to improve the interaction of exosomes with tumor cells. Briefly, exosomes were equipped with anti-epidermal growth factor receptor nanobodies (as a target ligand), using a lipid called glycosylphosphatidylinositol a linker (Kooijmans et al., 2016). Likely other lipids, due to their structural similarities to the exosomal membrane and hydrophobic interactions, could also be considered linkers to conjugate desired elements on the surface. In another study, maleimide-terminated 1,2-distearoyl-sn-glycero-3-phosphethanolamine-polyethylene glycol was conjugated to the surface of exosomes derived from MCF-7 cells. Although using maleimide-terminated 1,2-distearoyl-sn-glycero-3-phosphethanolamine-polyethylene glycol as a probe create limited specificity for binding and interaction, it has an insignificant effect on exosome biological activity or exosome morphology and makes exosomes useful for biomedical application, such as monitoring exosome-cell interactions. Using this approach can improve exosome functions without requiring any time-consuming or expensive methods (Di et al., 2019).

Adding lipid moieties, such as cholesterol, can reinforce the morphology of lipid bilayers and increase their rigidity. For example, using nucleolin-targeting aptamers covalently bound to cholesterol-polyethylene glycol in dendritic cells increased the rigidity of the lipid bilayer of nanovesicles produced by extrusion to target cancer cells. The treatment efficiency of these engineered nanovesicles was assessed by loading Paclitaxel both *in vitro* and *in vivo*, and a cytotoxicity assay confirmed the drug efficacy of this new nanovesicle delivery vehicle (Wan et al., 2018).

Apart from using lipids as linkers, their combination with other molecules can improve cellular uptake and exosomal cargo release in the cytosol. For example, conjugating commercially cationic lipids (Lipofectamine, LTX) and pH-sensitive fusogenic peptide (a peptide composed of repeating sequences of Glu-Ala-Leu-Ala) with exosomes, facilitated their interaction with HeLa cell membranes. This approach was shown to significantly enhance the release of Dextran (artificially loaded into exosomes). Negatively charged cationic lipids helped to accumulate fusogenic peptides, which itself increased the disruption of the endosomal membrane (Nakase and Futaki, 2015).

As mentioned, functionalizing exosomes with lipids can improve their targeting to a particular location in the body, which is important for therapeutic systems. Exosomes derived from the 3T3 mouse embryonic fibroblasts and A549 lung cancer cells were fused with synthetic lipids, including (1,2-dioleoyl-3-trimethylammonium-propane), (1,2-dipalmitoyl-sn-glycero-3-phosphocholine), (1-Palmitoyl-2-oleoyl-sn-glycero-3-phosphocholine), and (1-Palmitoyl-2-oleoyl-sn-glycero-3-phosphoglycerol) using extrusion. Then, by loading siRNA into these engineered extracellular vesicles, they were taken up by lung cancer cells with negligible cytotoxicity (Jhan et al., 2020). So, decorating exosomes with various lipids may improve targeting of brain cells. For example, in a similar study, to improve the properties of extracellular vesicles, such as tumor targeting and circulation time, exosomes derived from N2a cells and platelets were decorated with phospholipid 2-Dimyristoyl-sn-glycero-3-phosphoethanolamine (1,2-Dimyristoyl-sn-glycero-3-phosphoethanolamine and 1,2-Distearoyl-sn-glycero-3-phosphoethanolamine)-PEG-micelles (Kooijmans et al., 2016).

**FIGURE 7:**
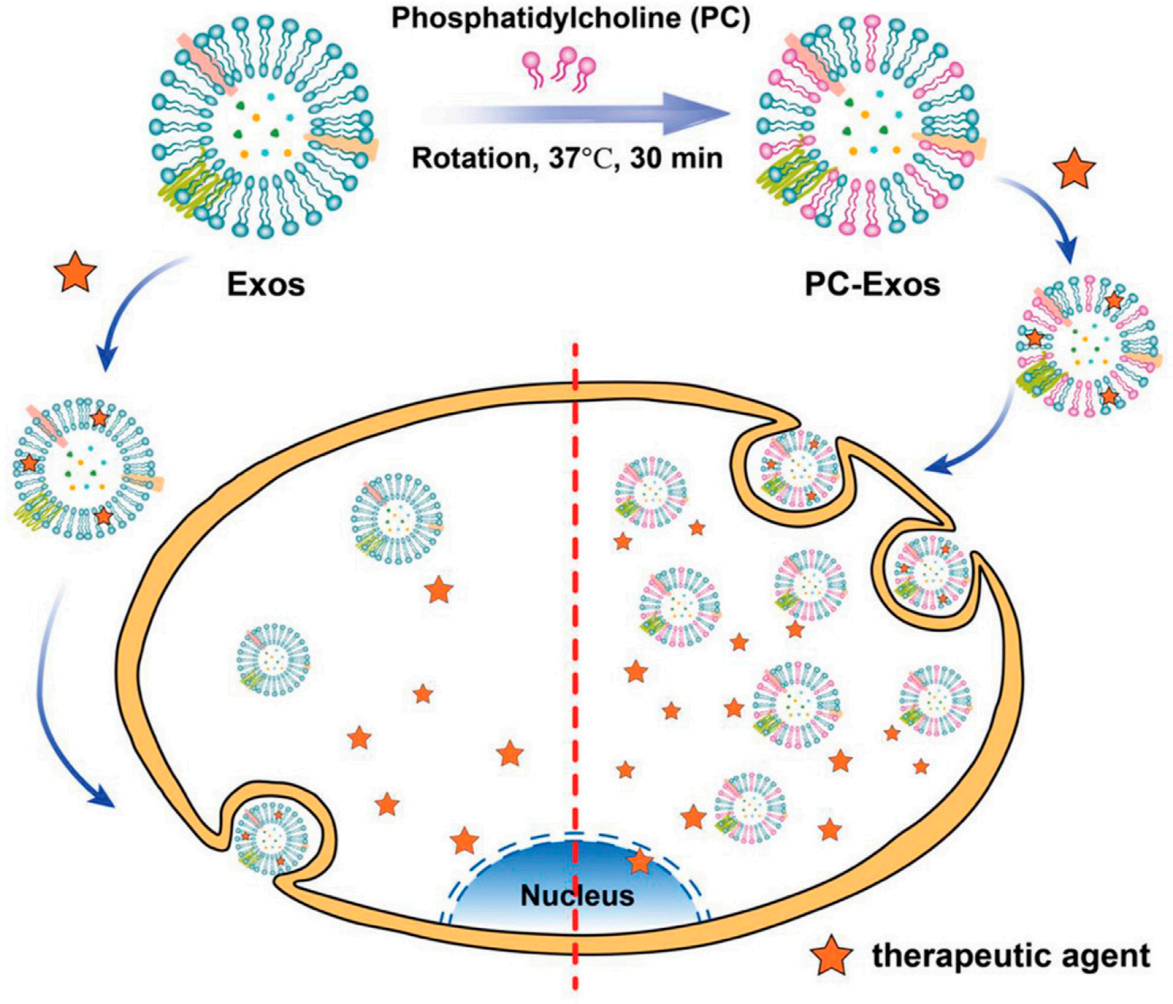
A schematic of engineered exosomes using phosphatidylcholine to improve the target uptake (Reproduced from Zhan et al., 2021).

Phosphatidylcholine is a potential moiety to improve the target uptake and circulation time of exosomes. Notably, exosomes derived from healthy cells have less tumor uptake efficiency than exosomes derived from tumor cells (Garofalo et al., 2019). Inspired by this phenomenon, reticulocyte-derived exosomes isolated from the serum of healthy mice were decorated with phosphatidylcholine molecules by mixing phosphatidylcholine molecules with exosomes, followed by ultracentrifugation. The *in vitro* studies of newly engineered phosphatidylcholine exosomes did not show changes in physical and biological properties, biocompatibility, or drug loading capacities. Phosphatidylcholine-Exos also showed an increase in uptake **(** Figure 7 **)** by the human glioblastoma U87 cells and breast cancer MDA-MB-231 cells (Zhan et al., 2021). Based on these studies, it can be expected that decorating exosomes with desired lipid ligands can make them a potential tool for drug delivery systems.

Another potential application might be altering the level of cholesterol and ceramides in the membrane to modulate exosome fluidity and prevent the spread of cytotoxic proteins, such as tau and amyloid β. In contrast, it might be possible to modulate the concentration of these lipids to increase membrane fluidity. For example, exosomes loaded with lysosomal enzymes might be able to trap cytotoxic proteins more efficiently for removal from the brain to treat Alzheimer’s disease.

### 4.2 Bioinspired and hybrid liposome-extracellular vesicle engineering approaches

Toward engineering EVs to endow them with enhanced functionalities, some work has been conducted that incorporates liposomes directly with EVs, or so-called “EV-liposome hybrids.” In a similar vein, EV compositions have been used to design liposomes with bio-inspired properties that mimic properties *in vivo*. These approaches have been widely explored in the context of adding specific surface markers but also recently investigated to include lipid composition that expands the realm of potential (synthetic) lipids to specifically modulate functionality, hydrophobicity, as well as cargo loading for therapeutic delivery. Liposomes, which are mono-layer or bilayer phospholipid vesicles containing an aqueous core, have been widely used as nanocarriers in drug and vaccine delivery systems for decades (Sercombe et al., 2015). Indeed, this hybrid EV-liposome approach uses liposomes as a foundation of biodegradable phospholipids and, at the same time, makes EVs more amendable to synthesize the developed nanovesicles (Lu and Huang, 2020). For example, exosomes derived from HEK293FT embryonal human kidney cells were isolated and, after fusing with liposomes, became suitable to encapsulate plasmids to induce CRISPR/Cas9 gene expression in human mesenchymal stem cells for gene therapy. Considering the potential toxicity of liposomes, hybridizing them with exosomes made them capable of entering the mesenchymal stem cells for gene expression. For this purpose, after incubating exosomes with liposomes and pEGFP-C1 plasmids, hybrid exosomes containing plasmids were produced after 12 h at 37°C (Lin et al., 2018).

Thermosensitive liposomes fused with engineered exosomes derived from CT26 murine colorectal cancer cells with overexpressed CD47 helped evade the immune system and increase blood circulation time. This hybrid system was shown to effectively target tumors *in vivo* and *in vitro* (Cheng et al., 2021). After genetically engineering fibroblasts, released exosomes enriched in CD47 were fused with thermosensitive liposomes by freeze-thaw (Cheng et al., 2021). The approach not only modulates the lipid composition of the system but enables the application of thermosensitivity to EV hybrids via the liposomal constituents.

Serum deficiency can induce mesenchymal stem cells to produce exosomes enriched in unsaturated and long-tailed cardiolipins (as well as other proteins) and be more active in delivering small interfering RNA to neurons. Inspired by this effect, artificially synthesized exosomes were designed using liposome compartments, dilysocardiolipin, and other proteins screened upon the induction of stress to modulate intercellular delivery (Haraszti et al., 2016). To synthesize the artificial exosomes, purified proteins (similar to proteins in exosome membranes) were palmitoylated to enhance their stability in the liposome membrane and conjugated by incubation (liposomes were synthesized using adioleoylphosphatidylcholine and cholesterol). The advantages of this bioinspired approach are higher yields and as well as efficient and straightforward cargo loading (Haraszti et al., 2016). Results showed that the vesicles were able to mimic the activity of exosomes, with the identification of dilysocardiolipin as playing a potentially notable role in trafficking activity and stressed conditions induced by serum deficiency. This work could underpin future studies whereby contributions of the lipid composition could be manipulated to alter function.

Only a few scientific groups have focused on lipid composition when synthesizing artificial “EV-like liposomes” or hybrid EV-liposomes. In one example, the effect of lipids, such as ceramide and cholesterol, on the human SOJ-6 pancreatic tumor cells was demonstrated by designing synthetic exosome-like nanoparticles mainly composed of sphingomyelin, ceramide, and cholesterol. The authors showed that synthetic exosome-like nanoparticles could increase tumor cell death by inducing sphingomyelinase activation and endoplasmic reticulum stress (Beloribi et al., 2012). By synthesizing exosomes, researchers used commercially available lipids inspiring the lipid composition of natural exosomes released from SOJ-6 in the human pancreatic cancer cells) (Beloribi et al., 2012). Most liposome-based exosomes have been studied on tumor cells, and there is an urgent need to use this method for the neurodegenerative disease to determine their impact on damaged brain cells. Thus, there is a massive gap regarding engineering exosomes with new lipid compositions, although the roles of lipids in equipping exosomes for cargo delivery is an area of active research.

## 5 Discussion

Initially, it was believed that EVs functioned solely in cellular debris degradation. However, recent studies have revealed that these nano-sized structures carry a diverse range of molecules, including DNA, RNA, proteins, lipolytic enzymes, and miRNA, serving various purposes, such as intercellular trafficking and targeting capabilities. As a result, characterizing and understanding the role of their composition is of the utmost importance to further elucidate their roles in the body. This is particularly due to their significant role in disease pathogenesis, inflammation, and immunity. While protein-focused studies have formed the vast majority of this work, it is clear that lipids, specifically their abundance, enrichment from parent cells, ratios, and density as they relate to EV interactions, are crucial to a holistic understanding of EV biology. While current techniques for lipid isolation and analysis have been introduced to enable scientists to investigate the roles of lipids in various scientific domains, it is clear that these approaches are still lacking with respect to their ease of use, availability, and cost. Therefore, new approaches to analytical characterization would help propel EV-based lipidomic research further in this burgeoning field.

It is clear that lipids possess diverse functional roles in the body, influencing different stages of EV biogenesis and altering their metabolism and distribution. Indeed, variations in lipid composition have been observed in the membranes of EVs derived from different cell types, while various studies have established a relationship between lipid enrichment in EVs and factors such as head group charge, fatty acid tail length, and saturation. These findings indicate the existence of lipid diversity changes in EVs based on their size and cell of origin and emphasize the importance of considering multiple exosome properties for accurate data interpretation. Furthermore, given that lipids play a crucial role in EV generation and release from their parent cells, understanding the impact of lipid structure on cell membranes can shed light on the role of lipids in the EV membrane and facilitate advancements in EV biogenesis through various techniques. This could be highly relevant for applications requiring high-throughput EV production in the future.

As described in this review, changes in the lipid composition of EV membranes have been linked to pathological conditions, including Alzheimer’s disease and cancer. These changes extend beyond overall lipid alterations and involve specific subclasses, such as ceramide species, which have been implicated in cancer-related processes. In the context of neurodegenerative diseases, the interaction between exosomal membrane lipids and toxic proteins, such as tau, underscores the potential role of exosomal lipids in disease development and their relevance in identifying new targets for therapeutic intervention and diagnostics. At the same time, EVs derived from the nervous system exhibit diverse lipidomic arrays, encompassing various cell types (e.g., neural stem cells, neurons, astrocytes, microglia, oligodendrocytes, Schwann cells, and endothelial cells). The sorting and enrichment of lipids in the EV membrane differ for each cell line and cell type, underscores their significance in EV production and their implications for lipid-focused studies. Notably, exosomal lipid imbalances hold potential as disease indicators in biological samples like urine or blood, offering promising avenues for clinical diagnostics. As a result, gathering extensive information on lipid changes in EV membranes under different conditions allows for the creation of patterns or lipidomic maps, which can greatly enhance disease diagnosis and monitoring capabilities. In the future, it can also be expected that manipulating EV lipids hold the potential to develop novel targets from an intervention and therapeutic perspective.

In this review we have provided an overview of exosomal lipids as both structural components and functional elements in EVs. However, further research is needed to fully understand the mechanisms by which EV lipids affect biological systems. Understanding how cells regulate the lipidomic array of EV lipids will be crucial for designing improved *in vitro* and bio-industrial production of EVs. Moreover, the lipid composition of EVs and its changes in different diseases, particularly in neurodegenerative diseases and cancer, hold potential for both therapeutic and diagnostic purposes. Generating complementary research data and a lipidomic data bank that incorporates changes in EV lipids specific to different diseases would aid in the clinical detection of pathological conditions, which complementary development of new lipid analysis techniques would further open up the potential in this field. From both an EV engineering and physiological perspective, the precise manipulation and rearranging of EV lipids to control their behavior still requires extensive research. This includes studying the spread of cytotoxic proteins between brain cells and exploring the potential to prevent the progression of Alzheimer’s disease through changes in EV lipids. Improving laboratory techniques and developing cell lines specifically designed to produce EVs with desired lipid arrays will facilitate a more precise investigation of the roles of EV lipids in Alzheimer’s disease and other conditions.

In conclusion, EV lipids play significant roles beyond their structural functions, making them important elements in the design of “smart” cellular compartments. The distinct lipid content observed in EV, particularly in brain cell-derived exosomes, can be harnessed to regulate their functionalities for potential disease treatments. The consideration of EV lipids in future studies, along with further research on functionalizing EVs, holds great promise for enhancing their diagnostic and therapeutic properties.
